# Plugging Teeth over Exposed Nerves

**Published:** 1854-07

**Authors:** 


### Dental Abstracts.

### *Plugging Teeth over Exposed Nerves.*

The January No. of the News Letter for '54, has an article on this subject from J. Lee, M. D., D. D. S., from which we take the following:

In excavating, when near the pulp, I excavate carefully each side, and turn off the covering if diseased; but in the teeth, as in the soft parts, the surgeon will find all around gangrenous, but the more vital parts healthy, and covered with sound parts. If so, let them alone, and fill your tooth, but you may, and *often* will find the floor of the cavity diseased; with a sharp edged instrument dexterously turn it off; having carefully cleaned every portion of decay from the diseased cavity, which can generally be done without wounding the nerve, introduce the foil, so as to cover the nerve with a smooth piece, probe down the sides strongly, and if they are well packed, you can go over the nerve with little pain, and your plugging will sueceed in nine out of ten cases. I have rarely failed. There is another condition to be noted ; having unco ered the nerve, it protrudes into the cavity: with a sharp excavator, I excise the protruding portion, and apply sulph. morphine on cotton, but this is not matetial.

I generally pass on to another part of the mouth, and can, in an hour, return to this, and plug it securely. It is absolutely necessary to success in this operation to give the vessels time to contract, and, theretore, the plugging must be delayed until they cease to bleed.

Teeth treated in this manner retain their vitality, while those from which the nerve has been removed, are sooner or later thrown off by the system. This mode of operating, I think better than perforating with a drill ; it, however, does not preclude that operation, if afterward required.

While on the subject of filling teeth, I would bear testimony to the use of two materials, and only two, to be used. In strong, sound teeth, gold is incontestably the best ; but there are several classes of decayed teeth, especially those affected with what has been termed white rot, where tin is superior to gold in arresting their decay. In plugging with it in these cases, I am not solicitous to keep it dry, as I believe the oxide of tin possess the quality of arresting the disease—certain it is that in the same mouth, with every care, I have had the disease to progress around gold plugs until they became loose, while the other teeth which I thought valueless, and were filled with tin, were preserved. Frequent observations of this have led me to recommend tin to my patients, and to use it with success in cases where I could not hope to succeed with gold.

There is yet another advantage in the use of tin over gold in such teeth. Their parieties are weak, and wou'd not bear the force necessary to consolidate a gold filling, while the utmost consolidation of tin is made with but comparatively little pressure. Tin is valuable as a plugging for decidous teeth—saves parents many sleepless nights, and retains in children's mouth's those teeth until the permanent are ready to come forward. I have seen the amalgam of tin and silver used for this purpose, by men who ought to have known better; and I have seen much suffering and some deformity from its use, causing salivation and the loss of the rudiments of the permanent teeth.

J. Lee, M. D., D. D. S.

---

Our Dental periodicals come to us filled with valuable and interesting matter, and we are sorry that we shall be able to select but few abstracts for this number of the Register.

That which perhaps at this time most interests the Profession is the use of sponge or crystal gold. We have given in the present number an article from the pen of Dr. Dwinelle, on this subject. The experiments instituted by this gentleman appear to be very conclusive, as regards its utility in plugging teeth.

The opinion we sometime since expressed, we have no reason to change. We still think it invaluable for many cases. Yet we think that for small cavities, and particularly approximal, good foil is most reliable—not but that experience may render it as valuable for even these cavities as any others.

We think that much care is necessary in its introduction, packing and consolidating, as it is introduced. We cut it into small blocks about the size of our blocks made with foil for filling and introduce it in the same manner, and where the cavity is large enough to permit the use of a blunt-pointed plugger, we are enabled to use it very satisfactorily.

We have used it in connection with foil, satisfactorily, only in the following cases: After having put in as much of the crystal gold as we supposed necessary, and by the use of a sharp-pointed condenser we find an imperfection; we then make an opening and fill up with foil. In such cases the foil appears to readily unite with the crystals; and we think such places are more certainly filled up and with less waste than with the crystal.

In the April number of the American Journal of Dental Science, we have an article on sponge gold by A. A. Blandy, M. D., D. D. S., and give his experiments in the use of the article:

That the experiments might be fair and thorough, the use of foil was, for the time, laid wholly aside, and the crystalline sponge substituted in all cases, no matter what tooth or the locality of the cavity in it to be filled. At first considerable difficulty was experienced in the working and management of the material, our instruments being sharp or wedge-pointed carriers, adapted only for the introduction of the foil, whereas for the introduction of the crystalline sponge, blunt concave-pointed instruments, roughened on the surface, are for the most part, better suited for introducing and consolidating the metal. If the crystals are separated from the spongy mass before they are brought firmly in contact with each other, they escape into the mouth and are lost, and to prevent which, when the instruments commonly employed are used, requires a great deal of tact and skillful management on the part of the operator. But this inconvenience and loss, decreased day by day, as the operators acquired experience and familiarity in the use of the article, until finally beautiful fillings were made with it even in the approximal surfaces of front teeth, but I am compelled to say that in the latter class of fillings such result was only obtained at the expense of a greater amount of time and labor than is required with foil, and also with considerable more waste of material.

When the cavity in the tooth is deep, great care is necessary to prevent choking the orifice, as the crystals unite as soon as they are brought firmly in contact with each other. When this happens it is with great difficulty that an instrument can be forced through the incrustation. The process of consolidating the gold, therefore, should be commenced at the bottom, and against the walls of the cavity. Unless this is done, it will be impossible to give to every part of the filling absolute solidity.

Again, if a sufficient quantity of the sponge to fill the cavity is not introduced before the mass is completely consolidated, it is almost impossible to add more, if the surface of that which was first introduced has become covered with moisture, unless the walls of the remaining portion of the cavity furnish sufficient mechanical support for its retention, or the gold already introduced presents a surface with one or more deep depressions capable of holding the fresh supply. This of course, need not occur in filling a cavity in the grinding surface of a tooth, but in the approximal it is particularly liable to happen. For filling a cavity in the grinding surface of an inferior molar or bicuspid, this preparation of gold is superior to any foil I have ever used.

In the April number of the News-Letter, we have an article on crystalized and sponge gold, by Dr. C. W. Ballard, No. 858 Broadway, N. Y., and give the following from his article on its use, and coincide with the Doctor, particularly in relation to its use for compound cavities:

These preparations are admirably calculated for filling compound cavities. Where the decay has extended to more than one side or surface of the tooth, such cavities can be filled and the form of the tooth restored with greater ease and security than with foil. The gain here is in the time, labor, and in the durability of the operation, as also in the occasional saving of tooth substance, in consequence of the diminished necessity of using the file.

Another advantage yet unmentioned is no slight one. It is always ready to be put into the cavity; it has only to be broken into small pieces of suitable size. Hence, the time spent in cutting, rolling, folding or twisting the foil, can be more profitably employed.

To sum up, its claims for professional favor, are: 1st, a saving of labor, of which most men are not too fond. If any are, they have 2d, a saving of time, and can consequently accomplish more in a day if they wish. 3d, a saving of tooth substance, in fact, of teeth. It is for this object we practice. 4th and last, and greatest, a better operation when completed.

I now propose to notice some objections I have heard raised against its adoption. They are more fallible than numerous; some are very trifling, though seriously urged, viz: 1st, *it is too expensive ;* 2d, there is too great waste; 3d, it will look badly in a front tooth when the walls of the cavity are composed only of enamel ; 4th, a dentist will have to learn to fill teeth over again ; 5th, an entire change of instruments will be required.

When I state that these are all the objections that have come to my ear, and I have heard the opinions of quite a number of dentists expressed, I believe many who may read this article, and who have hitherto considered the new preparation a humbug, will at once give it some attention. I most earnestly beg of them to do so, and hope they will give the profession the benefit of their experience. As the above objections have been seriously urged, I will endeavor to give them serious and satisfactory refutation.

As to the first, the cost per ounce is about the same as the best quality of gold foil. The advantages enumerated in the foregoing pages render it the cheapest in the end. 2d, in this respect there is a vast difference in the preparations offered for sale in this city. The one made by White, of Utica, is the one I have used the most extensively; it is darker colored, less plastic and less tenacious than the article made by J. A. Watts & Co., of the same place ; it crumbles more readily, and on that account gives trouble while filling the superior teeth. Where it can be used with facility, however, it possesses all the advantages over foil that I have enumerated, though not in as great a degree as the preparation of Watts. The latter is much more plastic and tenacious. It does not waste any more than foil, and I am satisfied that it will prove in any and every case better than the best foil.

The difference in color is sufficient to answer objection No. 3; Watts' preparation being near the shade of Abbey's foil, if anything, a shade lighter, and less brilliant when not burnished, consequently less apt to be noticed through the enamel. As to objection No. 4, I can only state my belief that a good operator, will, after a little practice, be able to work it with more ease than he can foil, and with greater service to the patient, and as for the poor operator, it will doubtless be greatly to his patient's benefit, the very first time he uses it instead of foil; in either case, the art of using it must be acquired, no matter at what expense of time, labor and money, if its use is going to benefit our patients. And now for No. 5. The ordinary round-pointed pluggers, with grooves filed across their edges in different directions so as to leave a number of small, well-defined points, are all that will be required for external cavities; for approximal cavities, we have only to combine the rough, condensing surface with an instrument sufficiently small as to allow of its being introduced between the teeth and into the cavity.

The April number of the News-Letter also contains an article from Dr. E. Townsend, on the use of crystal gold, in which he speaks of it in a commendable manner, giving some objections; also some of its advantages. Space forbids, as we should like, a publication of this article.

The March number of the Dental Recorder has a leading editorial on the "relation between the teeth and beard", from which we take the following:

We have long been of the opinion that the almost universal custom in this country of shaving the beard is fraught with more mischief to health and vigor of constitution than most people are willing to admit. We have altogether eschewed the use of the razor in our own case, save on the upper lip, and only tolerate it here out of a decent respect to popular opinion, and an individual dislike to the *mustache*, from certain unpleasant associations. But we do wish from our heart that the custom of shaving at all were entirely abandoned, believing as we do that it is decidedly a *barbarous* one.

And, individually, we are glad that the matter has been taken in hand by certain independent thinkers and writers who seem determined to bring about a real hirsute reformation.

That diseases of the throat, larynx, bronchia, etc., are often relieved, if not entirely prevented by withholding the razor from the face and throat, is too well known to be considered much of a novelty, our readers will agree.

And further in alluding to the effect of shaving on the teeth, the Dr. remarks:

Here then at the outset, we have this singular fact that precisely where the habit of shaving the face is most generally adopted and practiced, there is confessedly the greatest amount of suffering from diseases of the teeth. This we believe to be the fact, and whatever may be thought of it in this respect, we can but regard it as a coincidence of some importance. We doubt not but our very seriousness in the matter will provoke a smile on the face of some of our readers who may come clean and smoothly shaven from the chair of their barber; but we ask them to explain this coincidence, together with other considerations which we herewith present, if there be no force in the argument.

The same number contains an article from the Boston Medical & Surgical Journal on the same subject, which takes ground against the practice of shaving. We think indeed from various indications we see around us, and expressions of the press, that the sale of razors will be very much injured.

